# The Medical Department of Michigan and Our Cotemporaries

**Published:** 1855-05

**Authors:** 


"*The Medical Department of Michigan and our Cotemporaries.*"—We refer our readers to an article under the caption above, which we quote from the "Peninsular Journal" for April. It has enough of spice in its composition to make it eminently readable, and we do not like to undertake a reply to it, without doing full justice to our confrére, by giving our readers every opportunity to understand the issue.

The "Peninsular" assumes that the action of the Legislature, establishing a Chair of Homœopathy in the University, is to all intents and purposes void; inasmuch as, in its opinion, the regents will not follow out the instructions of the Legislature. We have no very extensive acquaintance with the organic law of Michigan. It may be that the people have placed it beyond the power of their representatives to control the affairs of their favorite institution. If this be so, nothing can be easier for the "Peninsular" than to quote, for the benefit of anxious outsiders, that clause, or provision in the Constitution, which gives to the Regents, only, the power to establish a professorship. And when the "Peninsular" shall have done this, we will concede that there will be no chair of homœopathy in the University of Michigan, until the Board of Regents in their wisdom create one. How long this may be, we do not undertake to say.

The principle, however, remains the same. The gentlemen of the Ann Arbor school are at all times answerable to a power outside of the profession —a power which may, or may not have those overflowing sympathies for legitimate medicine, which the "Peninsular" claims for the Board of Regents. And whatever our friend may say of the *quasi*-official character of the Board of Visitors, however many epithets it may heap upon the authors of the "Report" it so warmly denounces, it cannot conceal the fact that there now exists in Michigan a strong and determined opposition to the present management of the University—an opposition, which, if we may judge from the character of that portion of the public press which sustains it, will be likely at some early day to bring about the overthrow of its present officers.

We have no knowledge of the merits of the case between the Legislature, the Homœopaths, the Board of Visitors, the Detroit Free Press, and Prof. Allen on the one side, and the Faculty on the other—it is altogether probable that the latter may be entirely in the right, but can they carry on this fight without somewhat interfering with the quiet dignity of a professional life in the shire-town of Washtenaw (we believe) county—nay, would they fight at all without some strong motive—for instance a fear that some one was writing "mene, mene," etc., on the wall of their lecture room.

Let us assure the "Peninsular" that when it and the Buffalo Medical Journal, their editors, and the motives which actuate them, are alike forgotten, the principle for which we contend will still live—a medical school should recognize no control beyond that of the medical profession.

We have alluded to "motives," a favorite subject with the "Peninsular," for it always seems to think it unnecessary to reply to our arguments, so long as any question rests upon our motives. Is there no way that we can convince our friends that it is, most emphatically, none of their business what may be our motives. We will, if it wishes, acknowledge the soft impeachment, own up that our chiefest motive is the prosperity of the cause of independence of legislative control in medical education generally, and in the Buffalo School particularly, plead guilty to a general watchfulness over its interests, confess our personal, pocket-al, stake in the argument, but we still claim that the medical public will decide the question upon its own merits, without reference to the interest of either of the debaters in the matter.

And now we mean to go farther in that frankness and generosity on which our contemporary so compliments us. Speaking of us, he says, "if he had always been as frank in his expressions as he has now, he would have avoided some of those 'sore-headed rejoinders' of which he has so lively a remembrance, and which even still he seems to apprehend."

If our cotemporary has any distinct recollection of the particular "rejoinder" to which we alluded, he will recall certain epithets applied to us, such as we could not answer. Our readers felt no interest in Peninsular editorial opinions of ourselves, but farther than that, we believe that we have never yet made use of a similar style of argument—if we have, we pray to be forgiven! We had no alternative but to terminate the discussion. It has been opened again on more promising terms, and if the "Peninsular" is willing, it would afford us the greatest pleasure to debate with it any or all of the following questions, if we can do so without going farther into personality than that good-natured banter, which will make our prelections endurable to our readers:

1st. What will be the effect of free schools upon the *morale* of the profession?

2d. Should medical education be controlled in any degree by public sentiment, or by non-medical authorities?

3d. Is clinical instruction necessary to the efficiency of medical teaching?

4th. Is a fixed salary an incentive to activity in usefulness on the part of a medical teacher? And

5th. Does not the establishment of a free school by a state choke down all competition, and thus tend to the injury of the true interests of medical education?

Suppose that before settling who is victor in this discussion, we have a little direct and relevant argument upon the real points at issue. H.

